# Pituitary Enlargement and Hypopituitarism in Patients Treated with Immune Checkpoint Inhibitors: Two Sides of the Same Coin?

**DOI:** 10.3390/jpm13030415

**Published:** 2023-02-26

**Authors:** Sabrina Chiloiro, Antonella Giampietro, Antonio Bianchi, Sara Menotti, Flavia Angelini, Tommaso Tartaglione, Gian Carlo Antonini Cappellini, Federica De Galitiis, Ernesto Rossi, Giovanni Schinzari, Alessandro Scoppola, Alfredo Pontecorvi, Laura De Marinis, Maria Fleseriu

**Affiliations:** 1 Pituitary Unit, Department of Endocrinology and Metabolism, Fondazione Policlinico Universitario A. Gemelli, IRCCS, 00168 Rome, Italy; 2 Dipartimento di Medicina e Chirurgia Traslazionale, Università Cattolica del Sacro Cuore, 00186 Rome, Italy; 3 Dipartimento di Radiodiagnostica, Istituto Dermopatico dell’Immacolata IDI IRCCS, 00168 Rome, Italy; 4 UOC Oncologia, Ospedale Sandro Pertini, 00138 Rome, Italy; 5 UOC Oncologia, Istituto Dermopatico dell’Immacolata IDI IRCCS, 00185 Rome, Italy; 6 UOC Oncologia, Scienze Mediche e Chirurgiche, Fondazione Policlinico Universitario A. Gemelli, IRCCS, 00168 Rome, Italy; 7 UOSD Endocrinologia, Ospedale Santo Spirito, 00193 Rome, Italy; 8 Departments of Medicine (Division of Endocrinology, Diabetes and Clinical Nutrition) and Neurological Surgery, and Pituitary Center, Oregon Health & Science University, Portland, OR 97232, USA

**Keywords:** hypophysitis, autoimmune disease, chemotherapy, melanoma

## Abstract

Background: Immune checkpoint inhibitor hypophysitis (IIHs) is an emerging problem in cancer patients treated with immune checkpoint inhibitors (ICIs). We aimed to describe the clinical and molecular features of a multicenter series of IIHs. Methods: Demographic and clinical features were retrospectively collected for all cases. Anti-pituitary and anti-hypothalamus autoantibodies were also measured. Results: Nine patients were included. Six patients were treated with nivolumab and three with ipilimumab. Secondary hypoadrenalism was diagnosed in all patients. Pituitary MRI showed pituitary enlargement in two cases and no abnormalities in the other seven. Anti-pituitary antibodies were positive in 57.1% of cases and anti-hypothalamus antibodies in 85.7% of cases. Multidisciplinary treatments were established by a neuroendocrinologist and oncologists: all patients were treated with hydrocortisone replacement; ICI was withdrawn in two cases. At follow-up, hypoadrenalism persisted in all cases. Pituitary enlargement on MRI spontaneously recovered in the two affected patients. We found that the typical features of hypophysitis involved more frequently females and patients treated with ipilimumab. Conclusions: Although this study did not clarify if autoimmune secondary hypoadrenalism and ICI hypophysitis on brain imaging are two sides of the same disease, our preliminary data underline the need for molecular studies of IIHs and of autoimmune ICIs-related hypopituitarism.

## 1. Introduction

The recent development of immune checkpoint inhibitors (ICIs) has produced very promising results for the treatment of many malignancies, e.g melanoma; Hodgkin’s lymphoma; and head and neck squamous cell, urothelial, bladder, and non-small cell lung carcinomas [1,2,3], including significant antitumor activity [4]. Some ICIs, such as ipilimumab, inhibit the cytotoxic T-cell antigen-4 (CTLA-4), which acts early in the process of immune activation and increases the proliferation and activity of T cells [5]. Other ICIs, such as nivolumab and pembrolizumab, work as anti-PD1 drugs [6]. The immune checkpoints regulate the immune response involved in maintaining immunological homeostasis, thereby preventing the onset of autoimmune disease [7]. ICI can also cause a wide range of immune-related adverse events (IRAEs) [6,8], such as colitis, dermatitis, hepatitis, pancreatitis, nephritis, polymyositis, uveitis, toxic epidermal necrolysis, DRESS syndrome, hemophilia A, Tolosa–Hunt syndrome, Graves ophthalmopathy, thyroiditis, adrenalitis, and hypophysitis. The occurrence of IRAEs, including immunotherapy-induced hypophysitis (IIH), has been suggested as a positive predictor of ICI outcome [2]. Other endocrine-specific autoantibodies have been shown to play important roles in the pathogenesis of IRAEs as predictive markers for the early identification of ICI-induced endocrinopathies [9]. If IIH develops, patients need immediate treatment for hypopituitarism to avoid the possible withdrawal of ICIs [10]. Notably, inappropriate administration of immunosuppressive glucocorticoids (GCs) may negatively affect the patient’s prognosis. Over the last few years, the occurrence of IIHs has increased. There is a higher prevalence in patients treated with anti-CTLA4 [11] and with anti-CTLA4 plus anti-PD1 [10,12]. Furthermore, recent evidence suggests that the occurrence of anti-CTLA-4 induced hypophysitis may be independent of the dose (10 versus 3 mg/kg) and the number of treatment cycles [2,13,14]. The recurrence of hypophysitis after ICI rechallenge has also been described recently [15]. This contrasts with the traditional assumption that chronic, irreversible IRAEs are unlikely to recur [16]. 

In this study, we aim to describe our experience in the diagnosis, molecular investigation, and therapeutic management of a small series of patients with IIH.

## 2. Materials and Methods

A retrospective observational study was conducted on patients with IIHs.

Patients with IIH were diagnosed at “A. Gemelli Hospital,” at the “Istituto Dermopatico dell’Immacolata, IRCCS,” at the “Santo Spirito” Hospital, and at the “Sandro Pertini” Hospital,” all located in Rome. All protocol procedures were conducted at the Pituitary Unit of “A. Gemelli” Hospital, “Sacro Cuore” University. The local bioethics committee approved the study. All patients who were enrolled signed a consent form.

Patients entered this study based on the following inclusion criteria:

1. diagnosis of IIH;
2. age of 18 years or older;
3. availability of serum collected at the time of the IIH diagnosis.

### 2.1. Diagnosis of IIH and ICI Induced Hypopituitarism 

Patients with new onset of pituitary dysfunction during ICI treatment underwent contrasted pituitary magnetic resonance images (MRI). After ruling out focal lesions of the pituitary gland and/or stalk (such as pituitary and infundibular metastasis, non-secreting and prolactin-secreting pituitary adenomas, craniopharyngioma, germinoma, meningioma, glioma, pituitary apoplexy, physiological pituitary hypertrophy, or pituitary hyperplasia as a result of primary hormonal deficits) and causes of intracranial hypertension, IIH was diagnosed if there was a typical radiological finding of hypophysitis: pituitary enlargement and/or pituitary stalk swelling and/or absence of the posterior pituitary “bright spot” on T1-weighed (T1w) images [17].

The immunotherapy-induced hypopituitarism was defined in patients without the typical radiological finding of hypophysitis and after ruling out focal pituitary lesions and intracranial hypertension due to cerebral masses. 

### 2.2. Endocrine Assessment

According to our clinical protocol [18], patients receiving ICI underwent an evaluation of their clinical and past medical history before each treatment cycle, along with a hormonal evaluation of thyroid and adrenal functions. If new onset of symptoms such as asthenia, nausea, headache, decreased libido, amenorrhea, loss of appetite, edema, hypotension, hypoglycemia, tachycardia, or hyponatremia occurred, additional pituitary function tests were performed.

The assessment of pituitary function involved measuring hormone levels, including TSH (thyroid-stimulating hormone), free triiodothyronine, free tetraiodothyronine, adrenocorticotropic hormone (ACTH), cortisol, insulin-like growth factor-I (IGF-I), prolactin (PRL), luteinizing hormone, follicle-stimulating hormone, testosterone in males, and estradiol in females, as recommended by the Endocrine Society guidelines [19].

At the time of the IIH diagnosis, all patients underwent a clinical examination and/or laboratory testing to evaluate the presence of coexisting autoimmune polyendocrine syndromes (types I, II, III, and IV), Graves’ disease, type 1 diabetes, Addison’s disease, autoimmune thyroiditis (ACT), autoimmune hepatitis, autoimmune hypoparathyroidism, autoimmune atrophic gastritis, pernicious anemia, celiac disease, primary biliary cirrhosis, Sjogren’s disease, systemic lupus erythematosus, dermatomyositis, scleroderma, Behcet’s disease, Henoch–Schönlein purpura, and mixed connective tissue disease.

### 2.3. Neuroradiological Assessments

All magnetic resonance imaging (MRI) examinations were performed using a 1.5 T MR scanner (Optima HDxt, General Electric Healthcare) with a standardized imaging protocol that was based on thin-section images (2.6 mm thickness, 0.3 mm interspace). The protocol included coronal T2 W images (repetition time/echo time of 3300/120 ms), sagittal and coronal T1W images (TR/TE of 500/16 ms) before and after the intravenous administration of gadolinium (Multihance, Bracco at a dose of 0.1 mL/kg). For each patient, a dynamic contrast-enhanced sequence was obtained to study the pituitary gland (TR/TE of 450/16 ms, duration of 30 s, and 6 consecutive series).

Hypophysitis was anatomically classified [20] through pituitary MR images as follows:

1. Adenohypophysitis (AH) in cases with involvement of the anterior pituitary (pituitary enlargement) and without signs of involvement of the posterior pituitary.
2. Infundibulo-neurohypophysitis (INH) in cases with signs of infundibulum, pituitary stalk, and posterior pituitary involvement (thickness of the pituitary stalk and absence of the posterior pituitary bright spot on T1w images) without involvement of the adeno-pituitary.
3. Panhypophysitis (PH) in cases with involvement of the anterior pituitary, posterior pituitary, pituitary stalk, and infundibulum.

### 2.4. Immunofluorescence Technique for Anti-Pituitary and Anti-Hypothalamus Antibodies Detection

Anti-pituitary antibodies (APA) and anti-hypothalamus antibodies (AHA) were measured as previously described [21] using an indirect immunofluorescence method on monkey hypophysis slides (MHY) and monkey hypothalamus slides (MTH) provided by Biosystem S.A. [22]. The serum samples were tested for the presence of APA and/or AHA by their binding to the corresponding antigens on the monkey sections. The antigen–antibody complexes were then detected using a goat anti-human IgG conjugated with fluorescein isothiocyanate (FITC). Non-specific fluorescence was removed by adsorbing the IgG FITC with monkey serum. Serum samples were considered positive for APA and/or AHA at a dilution rate of 1:8, if a diffuse immunofluorescence pattern showing intracytoplasmic staining was observed in the majority of fields. Positive and negative controls were included in each assay.

## 3. Results

A total of nine patients met the inclusion criteria. Six of them were male (66.7%). The mean age of the patients at the time of onset of IIH was 59 years (standard deviation—SD: 16.8, minimum: 41, maximum: 83). Patients’ clinical features are summarized in Table 1 Seven patients had metastatic melanoma (all grade 4), one had metastatic lung adenocarcinoma (grade 4a), and another patient had metastatic kidney adenocarcinoma (grade 4). Six patients were treated with nivolumab and three patients with ipilimumab.

All patients experienced new-onset asthenia during the ICI treatment, which occurred after a mean of 7.5 ICI cycles (standard deviation: 5.9, minimum: 3, maximum: 19). Other symptoms were diarrhea in patient 1, fever in patient 2, hypoglycemia and hypovolemic shock in patient 3, and hypotension in patient 5 (Table 1). No patients complained of headaches or visual field deficits. Secondary hypoadrenalism was diagnosed in all patients, and no patient had a previous history of endocrine or autoimmune disorders. One patient (pt2) developed ICI-induced primary hypothyroidism before the hypophysitis.

**Figure 1:**
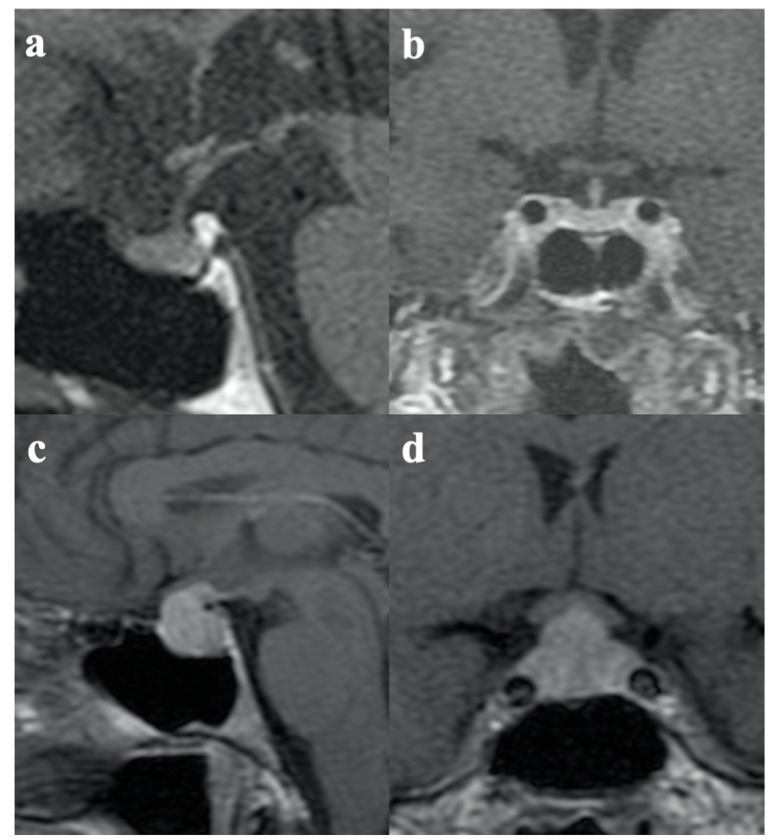
(**a**) Sagittal and (**b**) coronal T1-weighted images after contrast administration show normal pituitary and stalk volume and regular enhancement in a patient with ICI-induced hypoadrenalism. (**c**) Sagittal and (**d**) coronal T1-weighted images after contrast administration show typical features of hypophysitis: an enlarged pituitary gland and a thickened pituitary stalk with homogenous enhancement in a patient with IIH.

Pituitary MRI was performed in all patients at the time of diagnosis of secondary hypoadrenalism and showed a typical picture of anterior enlargement and swelling of the anterior pituitary with non-homogeneous contrast enhancement in two cases. In the remaining seven cases, no pituitary pathological features were detected by MRI (Table 2). Pituitary metastases were ruled out in all cases. Figure 1 shows a patient with the typical features of IIH Figure 1c,d and a case with immunotherapy-induced hypoadrenalism with normal radiological findings Figure 1a,b.

All patients were treated with replacement hydrocortisone for central hypoadrenalism. Patients 3 and 5 received parenteral hydrocortisone due to their severe clinical condition, which also required hospitalization. A multidisciplinary team of oncologists and endocrinologists evaluated the administration of high-dose immunosuppressive GC and ICI withdrawal, following clinical practice [18]. ICI was withdrawn in two patients (pt 3 and pt 5) until their clinical stability was achieved with hydrocortisone. High-dose immunosuppressive GCs was never prescribed due to the absence of either ophthalmological or neurological symptoms. However, prednisone was prescribed for a patient for the management of immunotherapy-induced diarrhea (pt 1). After a 3-month follow-up, pituitary MRI was performed in all cases. In the two patients with radiological pituitary enlargement (hypophysitis), spontaneous recovery was detected by MRI.

During the follow-up, all patients underwent periodically pituitary function screening: no worsening of pituitary function or recurrence of hypophysitis was detected. However, central hypoadrenalism persisted in all patients at the last examination. Six patients were considered partially or completely ICI-responsive, but one patient experienced cancer progression.

Seven (out of nine) patients developed central hypoadrenalism in the absence of radiological signs of inflammatory pituitary involvement, and two patients showed the typical radiological finding of hypophysitis. Table 2 shows that patients with radiological findings of hypophysitis were more frequently females, whereas patients with secondary hypoadrenalism without radiological findings of hypophysitis were more frequently males. 

**Figure 2:**
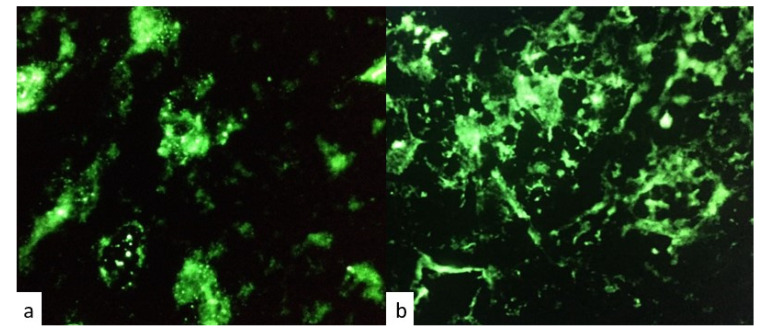
Immunofluorescence patterns for (**a**) positive anti-pituitary antibodies (APA) and (**b**) negative anti-pituitary antibodies (APA).

**Figure 3:**
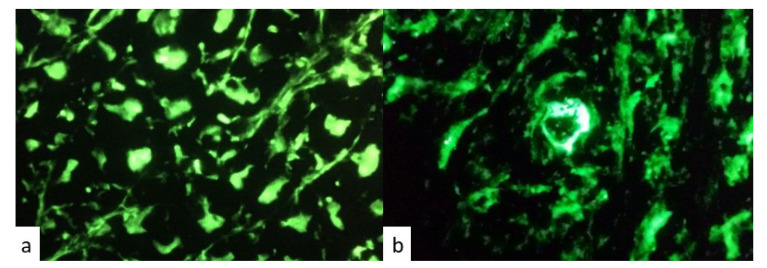
Immunofluorescence patterns for (**a**) positive anti-hypothalamus antibodies (AHA) and (**b**) negative anti-hypothalamus antibodies (AHA).

Positivity for anti-pituitary and anti-hypothalamus antibodies was detected in patients with IIHs and in those with ICI- induced hypopituitarism. Figure 2 and Figure 3 represent respectively positive and negative immunofluorescence for APA and AHA.

## 4. Discussion

Our case series suggests the presence of two distinct clinical phenotypes of IIH, and further differentiation may be beneficial. Hypophysitis was initially described in 1962 as a primary autoimmune disorder of the pituitary gland [23], but over the last decade, secondary hypophysitis has been reported due to the increasing number of cancer patients treated with ICI [24]. Among endocrine IRAEs, managing IIH remains challenging in clinical practice, as it requires balancing hypopituitarism treatment while avoiding ICI withdrawal. Additionally, avoiding high-dose glucocorticoids (GCs) is also crucial, as it may negatively impact the patient’s prognosis. A recent study by Faje et al., showed that patients treated with high-dose GCs had higher risks of ICIs failures and disease progression, and no improvement or resolution of IIH [25,26]. The European Society of Endocrinology clinical practice guidelines on hypophysitis emphasize that there is no clear evidence for the benefit of high-dose GC, with the possible exceptions of hypophysitis affecting the optic nerves [27]. Regarding the optimal timing of GC therapy initiation, a recent study developed a mathematical model to evaluate the comparative benefits of different GC administration schedules [28]. The authors suggested that the GCs treatment should be initiated precisely after the start of immunotherapy, as soon as the tumor volume begins to decrease under the effect of CTLA-4 inhibitors alone [28].

In previous clinical reports of IIH, secondary hypoadrenalism was observed in 50–73% of patients, and secondary hypogonadism in 85–100% of cases [9,29,30]. Interestingly, very few cases of anti-diuretic hormone (AVP) deficiency were reported, as IIH typically shows features of aden-hypophysitis, indicating an inflammatory involvement of the anterior lobe of the pituitary gland. The stalk thickening was observed less frequently in IIH, suggesting a lower frequency of inflammatory dysfunction in the neuro-pituitary in ICI-treated patients [31]. In our series, all patients had isolated central hypoadrenalism, and only one patient had primary hypothyroidism. The ICIs were temporarily withdrawn until GC replacement optimization was achieved, and immunosuppressive high-dose GC (over 40 mg Hydrocortisone/day) was never administered.

Patients with radiological evidence of hypophysitis were more frequently female, and patients with secondary hypoadrenalism without radiological evidence of hypophysitis were more frequently male. To our knowledge, our data are novel (versus previous studies depicted in Table 3); however, our results may have been influenced by the small sample size of our cohort and require further validation in a larger dataset.

Although females are generally more prone to develop autoimmune diseases, IIH affects males more frequently; the reason for this gender difference is not yet understood. It is believed that immune response to auto-antigens is weaker in males, resulting in a lower prevalence of autoimmune diseases. If this is partly due to stronger immune checkpoint activity in males, then immune checkpoint inhibition might trigger an excessive immune response in males and result in more secondary autoimmune disorders [31]. A large prospective study proved that endocrine-specific autoantibodies (such as thyroid, adrenal, and pancreatic antibodies) may be predictive markers for early identification and treatment of ICI-induced endocrinopathies [33].

The diagnostic criteria for IIHs are still not well defined. IIH is diagnosed as a new onset of hypopituitarism during ICI treatment, with or without the typical radiological findings of hypophysitis. The frequencies of IIHs vary greatly, ranging from 1.4–12% in clinical trials to 25% in real-world scenarios [9,34]. The incidence of IIHs is lower in cases with new onset hypopituitarism and radiological evidence of hypophysitis, and higher in cases diagnosed solely with new onset of hypopituitarism without such radiological evidence, as shown in Table 4 [35,36].

MRI abnormalities are seen in 81% of cases treated with anti-CTLA-4, whereas in 18% of patients with hypophysitis treated with anti-PD-1/anti-PD-L1, the initial enlargement of the pituitary returns to normal within weeks [27]. Interpretation of clinical and radiological features of IIHs can be influenced by various factors, including exogenous GC administration and hormonal imbalances related to the underlying oncological disease [18,26]. In some cases, radiological signs of IIHs may be mild and only noticeable when compared to previous MRIs [26]. The pituitary enlargement may resolve more quickly and even precede the clinical diagnosis of hypopituitarism [30].

Here, we compared the clinical and molecular features of ICI-induced hypopituitarism cases in patients with and without neuroradiological findings of hypophysitis. Some authors have proposed that hypophysitis and hypopituitarism induced by ICIs may represent different forms of “toxicity.” Our small case series showed that males were more frequently diagnosed with central hypoadrenalism without the typical imaging suggestive of hypophysitis as compared to females. Two cases with central hypoadrenalism and radiological IIH were treated with ipilimumab, a CTLA-4 inhibitor. It has been demonstrated that CTLA-4 is normally expressed by pituitary cells in both mouse and human models [7]. In a study of six patients treated with CTLA-4 inhibitors, autopsies revealed that CTLA-4 antigen was expressed by pituitary endocrine cells in all patients, but at various levels. The highest level of expression of protein was found in the patient with clinical and pathological evidence of severe hypophysitis, which was associated with T-cell infiltration, IgG-dependent complement fixation, and phagocytosis [37]. Similarly, a murine model showed that CTLA-4 inhibition leads to lymphocytic infiltration of the pituitary gland [7]. Genetic association studies have suggested that polymorphisms in CTLA4 can impact both autoimmunity and the toxicity of ICIs, and that germline polymorphisms in CTLA4 may influence susceptibility to IRAEs [38]. These findings may explain the different behaviors of central hypoadrenalism in patients treated with ipilimumab and nivolumab. In our study, anti-pituitary or anti-hypothalamus antibodies were present in both types of IIHs, which aligns with our previous study on primary autoimmune hypophysitis, where the prevalence of both APA and AHA was 68.4% [22]. A recent study of 62 cancer patients treated with ICIs showed that APA positivity was similar among the five patients who developed IIHs (80%) compared to those with an ACTH deficit (88.2%) [39]. Furthermore, two patients who developed anti-ACTH antibodies during ICI therapy have shown possible tumoral expression of ACTH, suggesting that ACTH deficit may be a paraneoplastic syndrome [40]. Visual impairments due to pituitary enlargement were more common in primary hypophysitis, which supports the concept that hypocortisolism in IIH is not primarily caused by compression of corticotroph cells [31], but possibly by the CTLA-4 pathway’s involvement. It has also been proposed that ICI-related hypophysitis may be associated with a paraneoplastic syndrome caused by ectopic expression of pituitary-specific antigens.

In addition to IIH, other novel clinical conditions caused by pituitary autoimmunity have recently emerged, known as “paraneoplastic autoimmune hypophysitis,” including anti-PIT-1 hypophysitis and paraneoplastic-autoimmune-isolated ACTH deficiency. These conditions have a shared underlying mechanism, where ectopic expression of pituitary antigen in cancer cells may evoke autoimmunity against specific pituitary cells and tumor immunity [40,41,42]. In isolated ACTH deficiency, ectopic ACTH expression in tumors can trigger autoreactive T-cell activation, and ICI administration can enhance autoimmunity, ultimately resulting in specific injury to corticotroph cells and ACTH deficiency [41]. Pituitary MRI in patients with isolated ACTH deficiency due to anti-PD-1 often shows a normal pituitary gland and low levels of ACTH or cortisol, which may persist for a long time after GC replacement therapy [43]. These entities may be on the same spectrum, highlighting the complexity of ICI-related hypophysitis in onco-immuno-endocrinology, and possibly explaining the large heterogeneity of clinical presentations in patients treated with ICIs [42,44].

The main limitations of this study are its small sample size and the lack of pathological examination of pituitary tissue, which makes it more difficult to define the molecular mechanisms involved in autoimmune secondary hypoadrenalism compared to IIHs. However, our preliminary data emphasize the need for further molecular investigation in a large cohort of patients. We highlight here the importance of a multidisciplinary management of patients diagnosed with hypopituitarism during ICI treatment, to rule out focal pituitary lesions or intracranial hypertension due to metastases, and to differentiate ICI-induced hypopituitarism from IIHs with a mass effect, which may require different management, treatment, and follow-up.

## 5. Conclusions

Although the results of this study do not clearly conclude on anything if autoimmune secondary hypoadrenalism and ICI hypophysitis seen on pituitary imaging are variations of the same disease, these preliminary data emphasize the need for further molecular research on IIHs and autoimmune ICI-related hypopituitarism, particularly central hypoadrenalism.

## Figures and Tables

**Table 1:** Clinical aspects of study cohort, endocrine adverse events during ICI, and clinical management.

	Tumor	TNM	Immunotherapy	Adverse Events/Grade	Pituitary Dysfunction	Pituitary MRI	Management/ Prescription
**Pt1**	Melanoma	T3bN3M1a	Ipilimumab 3 mg/kg	Asthenia (G1) Diarrhoea (G2)	Central hypoadrenalism	Hypophysitis	Oral hydrocortisone
**Pt2**	Melanoma	T4bN1bM1b	Ipilimumab 3 mg/kg	Asthenia (G1) Fever (G3)	Central hypoadrenalism	Hypophysitis	Oral hydrocortisone
**Pt3**	Melanoma	T2bN1acMx	Ipilimumab 3 mg/kg	Asthenia (G1) Hypovolemic shock (G3)	Central hypoadrenalism	Normal	I.V. hydrocortisone ICI withdrawn
**Pt4**	Melanoma	T3bN2bM1a	Nivolumab (240 mg every 15 days)	Asthenia (G1)	Central hypoadrenalism	Normal	Oral hydrocortisone
**Pt5**	Melanoma	T3bN2bM1b	Nivolumab (240 mg every 15 days)	Asthenia (G1) Hypotension (G3)	Central hypoadrenalism	Normal	I.V. hydrocortisone ICI withdrawn
**Pt6**	Lung adenocarcinoma	T3N3M1c	Nivolumab (240 mg every 15 days)	Asthenia (G1)	Central hypoadrenalism	Normal	Oral hydrocortisone
**Pt7**	Kidney adenocarcinoma	T4N1Mo	Nivolumab (240 mg every 15 days)	Asthenia (G1)	Central hypoadrenalism	Normal	Oral hydrocortisone

**Table 2:** Comparative analysis of demographic and clinical features of patients with ICI-induced central hypoadrenalism with and without the typical radiological signs of hypophysitis. Descriptive statistical analyses included finding the mean and standard deviation (SD) for continuous variables and absolute and relative frequencies for qualitative variables. Chi-squared and T-student tests were applied for univariate analysis. N: number.

	Central Hypoadrenalism		
	with Pituitary Enlargement on MRI	with Normal MRI	*p*-Value
**Gender** Female n, (%) Male n, (%)	2 (66.7%) 0	1 (33.3%) 4 (100%)	0.05
**Mean age at tumor diagnosis (SD)**	58 (22.6)	62 (18.7)	0.826
**Tumor type** Melanoma n, (%) Kidney n, (%) Lung n, (%)	2 (40%) 00	3 (60%) 1 (100%) 1 (100%)	0.571
**Previous treatments** None n, (%) Vemurafenib n, (%)	0 (0%) 1 (50%)	4 (100%) 1 (50%)	0.333
**Type of immunotherapy** Nivolumab n, (%) Ipililumab n, (%)	2 (100%)	5 (100%) 0	0.04
**Mean number of ICI administration (SD)**	3 (1)	9 (7)	0.394
**Mean age at central hypoadrenalism diagnosis (SD)**	58.5 (23.3)	63.7 (18.3)	0.774
**APA** Positive n, (%) Negative n, (%)	2 (50%) 0 (0%)	2 (50%) 3 (100%)	0.286
**AHA** Positive n, (%) Negative n, (%)	2 (33.3%) 0 (0%)	4 (66.7%) 1 (100%)	0.714
**Mean neutrophil count (SD) × 10^3^/mcgL**	4.6 (3)	5 (0.1)	0.873
**Mean lymphocytic count (SD) × 10^3^/mcgL**	1.96 (1.5)	2.28 (0.7)	0.808
**Mean neutrophil/lymphocytes ratio (SD)**	4.1 (4.7)	2.3 (0.8)	0.647

**Table 3:** Gender differences in IIH and ICI-induced hypopituitarism. Descriptive statistical analyses included absolute and relative frequencies for qualitative variables. Chi-squared test was applied for univariate analysis.

Authors (Ref)	Gender	IIH-Diagnosed on MRI	IIH Diagnosed with Hypopituitarism	*p*-Value
Albarel et al. [1]	M	**8 (47%)**	**9 (53%)**	0.613
	F	**4 (44.4%)**	**5 (55.6%)**	
Faje et al. [2]	M	**15 (50%)**	**15 (50%)**	0.999
	F	**2 (50%)**	**2 (50%)**	
Min et al. [23]	M	**10 (34.5%)**	**19 (65.5%)**	0.387
	F	**5 (45.4%)**	**6 (54.6%)**	
Ryder et al. [32]	M	**5 (31.2%)**	**11 (68.8%)**	0.873
	F	**4 (33.3%)**	**8 (66.7%)**	
Study cohort	M	**0**	**4 (100%)**	0.05
	F	**2 (66.7%)**	**1 (33.3%)**	
Whole cohort of patients	M	**43 (68.3%)**	**68 (71.6%)**	0.392
	F	**20 (31.7%)**	**27 (28.4%)**	

**Table 4:** Representative studies described cases of hypophysitis during ICI, classified according to different diagnostic criteria. * Brain imaging available for 49 pts; §: brain imaging available for 25 pts.

Authors (Ref.)	Year	Diagnosis	Number of Recruitment Centers	Study Duration (Months)	Immunotherapy Class	Number of Patients Enrolled	Number of IIH Diagnosed at MRI	Number of IIH Diagnosed Only with Hypopituitarism
Albarel et al. [1]	2015	Melanoma	1	74	anti-CTLA-4	131	12 (9%)	14 (10.7%)
Faje et al. [2]	2014	Melanoma	1	69	anti-CTLA-4	144	17 (11.8%)	17 (11.8%)
Jessel et al. [7]	2022		1	48	anti-CTLA-4 plus anti-PD1 Anti-PDL1 Anti-CTLA-4	490	23 (4.7%) *	65 (13%)
Min et al. [23]	2015	Melanoma	1	66	anti-CTLA-4	187	15 (8%)	25 (13.4%)
Kotwal et al. [30]	2022	Various Melanoma	1	Around 24	anti-PD1	656	16 (1.7%) §	7 (1.1%)
					anti-CTLA-4 plus anti-PD1	50		3 (6%)
					anti-CTLA-4	120		8 (6.7%)
					anti-CTLA-4 before/after anti-PD1	70		8 (11.4%)
Slovin et al. [9]	2013	Prostate cancer	9	44	anti-CTLA-4	71	1 (1.4%)	3 (4.2%)
Scott et al. [31]	2017	Melanoma	1	18	anti-PD1	103	3 (4%)	8 (10.8%)
					anti-CTLA-4 plus anti-PD1	59		
					anti-CTLA-4	15		
Ryder et al. [32]	2014	Melanoma	1	around 72	anti-CTLA-4	211	9 (4.3%)	19 (9%)

## Data Availability

The data presented in this study are available on request from the corresponding author.

## References

[1] Albarel F., Gaudy C., Castinetti F., Carré T., Morange I., Conte-Devolx B., Grob J.-J., Brue T. (2015). Long-term follow-up of ipilimumab-induced hypophysitis, a common adverse event of the anti-CTLA-4 antibody in melanoma. Eur. J. Endocrinol..

[2] Faje A.T., Sullivan R., Lawrence D., Tritos N.A., Fadden R., Klibanski A., Nachtigall L. (2014). Ipilimumab-Induced Hypophysitis: A Detailed Longitudinal Analysis in a Large Cohort of Patients with Metastatic Melanoma. J. Clin. Endocrinol. Metab..

[3] Chang L.-S., Barroso-Sousa R., Tolaney S.M., Hodi F.S., Kaiser U.B., Min L. (2019). Endocrine Toxicity of Cancer Immunotherapy Targeting Immune Checkpoints. Endocr. Rev..

[4] Takahashi Y. (2022). Onco-immuno-endocrinology: An emerging concept that links tumor, autoimmunity, and endocrine disease. Best Pract. Res. Clin. Endocrinol. Metab..

[5] Dillard T., Yedinak C.G., Alumkal J., Fleseriu M. (2010). Anti-CTLA-4 antibody therapy associated autoimmune hypophysitis: Serious immune related adverse events across a spectrum of cancer subtypes. Pituitary.

[6] Di Dalmazi G., Ippolito S., Lupi I., Caturegli P. (2019). Hypophysitis induced by immune checkpoint inhibitors: A 10-year assessment. Expert Rev. Endocrinol. Metab..

[7] Iwama S., De Remigis A., Callahan M.K., Slovin S.F., Wolchok J.D., Caturegli P. (2014). Pituitary Expression of CTLA-4 Mediates Hypophysitis Secondary to Administration of CTLA-4 Blocking Antibody. Sci. Transl. Med..

[8] Wright J.J., Johnson D.B. (2022). Approach to the Patient with Immune Checkpoint Inhibitor—Associated Endocrine Dysfunction. J. Clin. Endocrinol. Metab..

[9] Jessel S., Weiss S.A., Austin M., Mahajan A., Etts K., Zhang L., Aizenbud L., Perdigoto A.L., Hurwitz M., Sznol M. (2022). Immune Checkpoint Inhibitor-Induced Hypophysitis and Patterns of Loss of Pituitary Function. Front. Oncol..

[10] Langlois F., Varlamov E.V., Fleseriu M. (2022). Hypophysitis, the Growing Spectrum of a Rare Pituitary Disease. J. Clin. Endocrinol. Metab..

[11] Snyders T., Chakos D., Swami U., Latour E., Chen Y., Fleseriu M., Milhem M., Zakharia Y., Zahr R. (2019). Ipilimumab-induced hypophysitis, a single academic center experience. Pituitary.

[12] Barroso-Sousa R., Barry W.T., Garrido-Castro A.C., Hodi F.S., Min L., Krop I.E., Tolaney S.M. (2018). Incidence of Endocrine Dysfunction Following the Use of Different Immune Checkpoint Inhibitor Regimens. JAMA Oncol..

[13] Angelousi A., Chatzellis E., Kaltsas G. (2018). New Molecular, Biological, and Immunological Agents Inducing Hypophysitis. Neuroendocrinology.

[14] Abdel-Rahman O., El Halawani H., Fouad M. (2016). Risk of endocrine complications in cancer patients treated with immune check point inhibitors: A meta-analysis. Future Oncol..

[15] Park B.C., Jung S., Wright J.J., Johnson D.B. (2022). Recurrence of Hypophysitis After Immune Checkpoint Inhibitor Rechallenge. Oncologist.

[16] Johnson D.B., Nebhan C.A., Moslehi J.J., Balko J.M. (2022). Immune-checkpoint inhibitors: Long-term implications of toxicity. Nat. Rev. Clin. Oncol..

[17] Caturegli P., Newschaffer C., Olivi A., Pomper M.G., Burger P.C., Rose N.R. (2005). Autoimmune Hypophysitis. Endocr. Rev..

[18] Chiloiro S., Capoluongo E.D., Tartaglione T., Giampietro A., Bianchi A., Giustina A., Pontecorvi A., De Marinis L. (2019). The Changing Clinical Spectrum of Hypophysitis. Trends Endocrinol. Metab..

[19] Fleseriu M., Hashim I.A., Karavitaki N., Melmed S., Murad M.H., Salvatori R., Samuels M.H. (2016). Hormonal Replacement in Hypopituitarism in Adults: An Endocrine Society Clinical Practice Guideline. J. Clin. Endocrinol. Metab..

[20] Tartaglione T., Chiloiro S., Laino M.E., Giampietro A., Gaudino S., Zoli A., Bianchi A., Pontecorvi A., Colosimo C., De Marinis L. (2018). Neuro-radiological features can predict hypopituitarism in primary autoimmune hypophysitis. Pituitary.

[21] Chiloiro S., Tartaglione T., Angelini F., Bianchi A., Arena V., Giampietro A., Mormando M., Sciandra M., Laino M.E., De Marinis L. (2017). An Overview of Diagnosis of Primary Autoimmune Hypophysitis in a Prospective Single-Center Experience. Neuroendocrinology.

[22] Chiloiro S., Giampietro A., Angelini F., Arena V., Stigliano E., Tartaglione T., Mattogno P.P., D’Alessandris Q.G., Lauretti L., Pontecorvi A. (2021). Markers of humoral and cell-mediated immune response in primary autoimmune hypophysitis: A pilot study. Endocrine.

[23] Goudie R.B., Pinkerton P.H. (1962). Anterior hypophysitis and hashimoto’s disease in a young woman. J. Pathol. Bacteriol..

[24] Lupi I., Brancatella A., Cosottini M., Viola N., Lanzolla G., Sgrò D., Di Dalmazi G., Latrofa F., Caturegli P., Marcocci C. (2019). Clinical heterogeneity of hypophysitis secondary to PD-1/PD-L1 blockade: Insights from four cases. Endocrinol. Diabetes Metab. Case Rep..

[25] Faje A.T., Lawrence D., Flaherty K., Rn C.F., Fadden R., Rubin K., Cohen J., Sullivan R.J. (2018). High-dose glucocorticoids for the treatment of ipilimumab-induced hypophysitis is associated with reduced survival in patients with melanoma. Cancer.

[26] Min L., Hodi F.S., Giobbie-Hurder A., Ott P.A., Luke J.J., Donahue H., Davis M., Carroll R.S., Kaiser U.B. (2015). Systemic high-dose corticosteroid treatment does not improve the outcome of ipilimumab-related hypophysitis: A retrospective cohort study. Cancer Res. Commun..

[27] Husebye E.S., Castinetti F., Criseno S., Curigliano G., Decallonne B., Fleseriu M., Higham C.E., Lupi I., Paschou S.A., Toth M. (2022). Endocrine-related adverse conditions in patients receiving immune checkpoint inhibition: An ESE clinical practice guideline. Eur. J. Endocrinol..

[28] Siewe N., Friedman A. (2022). Optimal timing of steroid initiation in response to CTLA-4 antibody in metastatic cancer: A mathematical model. PLoS ONE.

[29] Byun D.J., Wolchok J.D., Rosenberg L.M., Girotra M. (2017). Cancer immunotherapy—Immune checkpoint blockade and associated endocrinopathies. Nat. Rev. Endocrinol..

[30] Faje A.T. (2016). Immunotherapy and hypophysitis: Clinical presentation, treatment, and biologic insights. Pituitary.

[31] Amereller F., Deutschbein T., Joshi M., Schopohl J., Schilbach K., Detomas M., Duffy L., Carroll P., Papa S., Störmann S. (2022). Differences between immunotherapy-induced and primary hypophysitis—A multicenter retrospective study. Pituitary.

[32] Ryder M., Callahan M., Postow M.A., Wolchok J., Fagin J.A. (2014). Endocrine-related adverse events following ipilimumab in patients with advanced melanoma: A comprehensive retrospective review from a single institution. Endocr.-Relat. Cancer.

[33] Labadzhyan A., Wentzel K., Hamid O., Chow K., Kim S., Piro L., Melmed S. (2022). Endocrine Autoantibodies Determine Immune Checkpoint Inhibitor-induced Endocrinopathy: A Prospective Study. J. Clin. Endocrinol. Metab..

[34] Kotwal A., Rouleau S.G., Dasari S., Kottschade L., Ryder M., Kudva Y.C., Markovic S., Erickson D. (2022). Immune checkpoint inhibitor-induced hypophysitis: Lessons learnt from a large cancer cohort. J. Investig. Med..

[35] Slovin S. (2013). Emerging treatments in management of prostate cancer: Biomarker validation and endpoints for immunotherapy clinical trial design. ImmunoTargets Ther..

[36] Scott E.S., Long G.V., Guminski A., Clifton-Bligh R.J., Menzies A.M., Tsang V.H. (2018). The spectrum, incidence, kinetics and management of endocrinopathies with immune checkpoint inhibitors for metastatic melanoma. Eur. J. Endocrinol..

[37] Caturegli P., Di Dalmazi G., Lombardi M., Grosso F., Larman H.B., Larman T., Taverna G., Cosottini M., Lupi I. (2016). Hypophysitis Secondary to Cytotoxic T-Lymphocyte–Associated Protein 4 Blockade. Am. J. Pathol..

[38] Chye A., Allen I., Barnet M., Burnett D.L. (2022). Insights into the Host Contribution of Endocrine Associated Immune-Related Adverse Events to Immune Checkpoint Inhibition Therapy. Front. Oncol..

[39] Kobayashi T., Iwama S., Sugiyama D., Yasuda Y., Okuji T., Ito M., Ito S., Sugiyama M., Onoue T., Takagi H. (2021). Anti-pituitary antibodies and susceptible human leukocyte antigen alleles as predictive biomarkers for pituitary dysfunction induced by immune checkpoint inhibitors. J. Immunother. Cancer.

[40] Kanie K., Iguchi G., Bando H., Urai S., Shichi H., Fujita Y., Matsumoto R., Suda K., Yamamoto M., Fukuoka H. (2021). Mechanistic insights into immune checkpoint inhibitor-related hypophysitis: A form of paraneoplastic syndrome. Cancer Immunol. Immunother..

[41] Bando H., Kanie K., Takahashi Y. (2022). Paraneoplastic autoimmune hypophysitis: An emerging concept. Best Pract. Res. Clin. Endocrinol. Metab..

[42] Takahashi Y. (2022). The novel concept of “Onco-Immuno-Endocrinology” led to the discovery of new clinical entity “paraneoplastic autoimmune hypophysitis”. Best Pract. Res. Clin. Endocrinol. Metab..

[43] Lin S.-H., Zhang A., Li L.-Z., Zhao L.-C., Wu L.-X., Fang C.-T. (2022). Isolated adrenocorticotropic hormone deficiency associated with sintilimab therapy in a patient with advanced lung adenocarcinoma: A case report and literature review. BMC Endocr. Disord..

[44] Yamamoto M., Iguchi G., Bando H., Kanie K., Hidaka-Takeno R., Fukuoka H., Takahashi Y. (2020). Autoimmune Pituitary Disease: New Concepts with Clinical Implications. Endocr. Rev..

